# Endometrial mesonephric-like adenocarcinoma: Clinicopathologic features, treatment, and outcomes

**DOI:** 10.17305/bb.2025.12993

**Published:** 2025-11-05

**Authors:** Shuping Yan, Yanpeng Tian, Mingyue Li, Xiaonan Wang, Jiayi Wang, Kuisheng Chen, Tianjiao Lai, Ye Zhang, Xiaoxiao Zhang, Yana Liu, Yuxi Jin, Xueyan Liu, Meng Mao, Qian Wang, Ruixia Guo

**Affiliations:** 1 Department of Pathology, The First Affiliated Hospital of Zhengzhou University, Zhengzhou, Henan, China; 2 Department of Obstetrics and Gynecology, The First Affiliated Hospital of Zhengzhou University, Zhengzhou, Henan, China

**Keywords:** Uterine cancer, mesonephric-like adenocarcinoma, clinicopathological features, prognosis, retrospective analysis, KRAS mutation

## Abstract

Endometrial mesonephric-like adenocarcinoma (MLA) is a rare subtype of uterine corpus endometrial carcinoma (UCEC) first described in 2016. The clinicopathological features, treatment options, and prognosis of endometrial MLA remain poorly understood. In this study, we retrospectively analyzed the clinicopathological characteristics, molecular features, treatment regimens, and outcomes of 11 patients diagnosed with endometrial MLA. The most prevalent symptom observed was postmenopausal bleeding. Notably, 78% (7 out of 9) of patients were diagnosed at advanced FIGO stages (II-IV), with four cases presenting with distant metastasis upon initial examination. Multivisceral metastases were identified in three cases, with lung metastases being the most common, occurring in 45% of patients. The median progression-free survival (PFS) was 16 months (95% confidence intervals: 6–26). All tumors tested negative for progesterone receptors (PR), while 91% of patients (10 out of 11) were negative for estrogen receptors (ER). Most patients exhibited positive immunohistochemical staining for “mesonephric-like” markers, including GATA-binding protein 3 (GATA-3), thyroid transcription factor-1 (TTF-1), and CD10. Furthermore, 91% of patients showed a wild-type p53 immunostaining pattern. Among the 11 patients, five underwent *KRAS* mutation testing, revealing *KRAS* mutations in all tested individuals (p.G12D in 2/5, p.G12A in 1/5, p.G12V in 1/5, and p.G13D in 1/5). These findings indicate that 78% of endometrial MLA patients were diagnosed at an advanced stage and suggest that this subtype may exhibit more aggressive behavior compared to endometrial endometrioid carcinoma. The consistent presence of *KRAS* mutations in patients who underwent testing highlights the potential role of *KRAS* in the initiation and progression of endometrial MLA, positioning it as a promising therapeutic target.

## Introduction

Uterine cancer ranks among the most prevalent gynecological malignancies worldwide, with its incidence rising each year [1, 2]. According to cancer statistics for 2023, uterine corpus cancer is the fourth most common malignancy among women in the United States, with nearly 66,200 new cases diagnosed and approximately 13,030 deaths in the United States [1]. In China, uterine cancer is the eighth most frequently diagnosed malignancy in women [3]. Approximately 90% of uterine cancers are classified as endometrial carcinomas (ECs) [4]. Recent trends indicate a rising incidence of ECs, often with an earlier onset age. Several risk factors have been identified for ECs, including obesity, nulliparity, elevated insulin levels, and the use of tamoxifen [5]. The predominant subtypes of ECs are endometrioid and serous carcinomas, which account for 80%–85% and 5%–10% of newly diagnosed cases, respectively [6]. A recent study identified novel and rare histopathological subtypes. McFarland et al. [7] reported in 2016 on seven cases of uterine cancers and five cases of ovarian cancers diagnosed as mesonephric-like adenocarcinoma (MLA). Subsequently, there has been an increase in reported cases of endometrial MLA, which correlates with a poor prognosis. Given that the pathogenesis of MLA is not yet fully understood, it is imperative to allocate resources to explore the clinical, pathological, and molecular features of MLA to enhance therapeutic guidance and improve prognostic outcomes.

In 2020, the World Health Organization (WHO) classified MLA as a novel pathological category of female genital tumors [8]. Currently, MLA is recognized as an exceedingly rare and diagnostically challenging subtype of ECs. Previous studies indicate that endometrial MLA comprises approximately 1% of EC cases [9]. Despite its low incidence, MLA has garnered international attention as a distinct entity within the spectrum of endometrial malignancies, predominantly affecting postmenopausal women. Clinically, endometrial MLA often presents with abnormal uterine bleeding, pelvic pain, or is discovered incidentally during imaging studies [10]. Endometrial MLA exhibits malignant characteristics, aggressive growth patterns, and a poor prognosis [7]. Pors et al. [10, 11] identified that patients with advanced-stage endometrial MLA had a worse prognosis, characterized by shorter progression-free survival (PFS) and overall survival (OS). Platinum- and taxane-based chemotherapy remains the standard first-line treatment for ECs [12]. However, advancements in targeted therapy and immunotherapy have improved outcomes for advanced and recurrent ECs [13, 14]. Currently, surgery is the preferred treatment for early-stage endometrial MLA. Nonetheless, due to the rarity of endometrial MLA, standardized and effective therapeutic options for advanced stages and recurrences remain lacking. Consequently, the prognosis for advanced-stage endometrial MLA is still unfavorable.

Accurate diagnosis of MLA necessitates a comprehensive approach involving clinical, radiological, and histopathological evaluations, including immunohistochemical analysis to distinguish it from other endometrial malignancies. Histologically, endometrial MLA presents various architectural patterns and cytological features, including tubular, solid, ductal, papillary, retiform, and sex cord-like structures [15]. Immunohistochemical profiling of MLA-associated markers typically reveals positivity for GATA binding protein 3 (GATA3), transcription termination factor 1 (TTF1), calretinin, and CD10 [10]. Notably, estrogen receptor (ER) and progesterone receptor (PR) are often negative. Molecular analyses have identified mutations in KRAS, NRAS, PIK3CA, and ARIDIA in MLA tissues [10, 16]. However, the clinicopathological features and pathogenesis of MLA remain poorly understood. Given the limited information available, further elucidation of MLA’s clinical behavior, optimal management strategies, and prognostic factors is necessary.

This study retrospectively investigates the clinicopathological features and prognosis of 11 patients diagnosed with endometrial MLA. By enhancing our understanding of the clinical characteristics, histopathological features, and treatment outcomes associated with MLA, we aim to improve diagnostic accuracy, tailor treatment interventions, and refine prognostic predictions for patients afflicted with endometrial MLA. Ultimately, this research seeks to provide valuable insights that may inform future investigations and clinical decision-making for patients with MLA.

## Materials and methods

### Study design

This study is a single-center retrospective analysis conducted by the Department of Obstetrics and Gynecology at the First Affiliated Hospital of Zhengzhou University. We enrolled 11 patients diagnosed with MLA, confirmed by preoperative biopsy or postoperative pathological examination. Hematoxylin–eosin (HE) and immunohistochemical (IHC) stained slides were evaluated by two experienced pathologists specializing in gynecological pathology at the Department of Pathology. All clinicopathological data were collected and analyzed.

### Clinicopathological data collection

Clinicopathological data were collected and recorded by designated gynecological oncologists. The data included age at diagnosis, primary symptoms, tumor biomarkers, pathological findings, tumor metastasis, type of surgery, adjuvant treatment, FIGO stage, presence or absence of recurrences, recurrence site, tumor progression, and survival outcomes. PFS was calculated from the date of diagnosis to the date of recurrence or death (if no recurrence occurred). Follow-up time was defined as the date of the last known follow-up or death.

### Immunohistochemical staining evaluation

All tissue samples were fixed in paraformaldehyde and embedded in paraffin. Paraffin-embedded blocks were sectioned into 4 µm slices. IHC staining was performed using an automated slide staining system (BenchMark Ultra, Roche) and the ultraView universal DAB detection kit. IHC staining results were evaluated by two experienced gynecological pathologists. Antibody information is presented in Table 1.

**Table 1:** List of antibodies

**Antibody**	**Manufacture**	**Cloning information**	**Cat No.**
Anti-ER antibody	Roche, Germany (RTU)	SP1	790-4325
Anti-PR antibody	Roche, Germany (RTU)	1E2	790-4296
Anti-GATA3 antibody	Jie Hao, China (RTU)	L50-823	GM-0091
Anti-CD10 antibody	Zhongshan Goldenbridge, China (RTU)	polyclonal	ZM-0283
Anti-TTF1 antibody	Maixin Biotech, China (RTU)	MX011	MAX-0677
Anti-PAX8 antibody	Zhongshan Goldenbridge, China (RTU)	OTI6H8	ZM-0468
Anti-AE1/AE3 antibody	Maixin Biotech, China (RTU)	AE1/AE3	Kit-0009
Anti-Vimentin antibody	Zhongshan Goldenbridge, China (RTU)	UMAB159	ZM-0260
Anti-P53 antibody	Zhongshan Goldenbridge, China (RTU)	DO-7	ZM-0408
Anti-P16 antibody	Roche, Germany (RTU)	E6H4	705-4713
Anti-Ki-67 antibody	Gene Tech, China (RTU)	GM027	GT209407

Abbreviations: ER: Estrogen receptor; PR: Progesterone receptor; GATA3: GATA-binding protein 3; TTF-1: Thyroid transcription factor-1; PAX8: Paired box 8.

### KRAS mutation test

DNA was isolated from 4 µm thick slices using a nucleic acid extraction kit (FFPE DNA, Omega Bio-Tek, USA). KRAS mutations were tested using the Human KRAS Gene Mutation Detection Kit (Amoy Diagnostics Co., Ltd., Xiamen, China). Protocols were executed according to the kit instructions [17, 18]. Quantitative real-time PCR was performed following established protocols from previous studies [19, 20].

### Ethical approval

Ethical approval was obtained from the Ethics Committee of the First Affiliated Hospital of Zhengzhou University (2023-KY-0563-001). Informed consent was obtained from all patients and/or their families.

### Statistical analysis

Clinicopathological data were summarized using descriptive statistics. Non-normally distributed variables were expressed as medians, while normally distributed continuous variables were reported as means ± standard deviation (SD). All statistical analyses were conducted using GraphPad Prism 10.1 software. Survival analyses were performed using SPSS version 26.0 and GraphPad Prism version 10.1. Kaplan–Meier curves were generated to estimate PFS, and two-sided 95% confidence intervals were calculated.

## Results

This study enrolled 11 patients, with a mean age of 62.18 ± 9.24 years (range, 48–74). Over half of the patients were classified as either overweight (*n* ═ 5, 25<BMI≤30) or obese (*n* ═ 1, 30<BMI≤40). All cases, except for cases 3 and 6, were classified according to FIGO stages. Among the staged cases, 78% (7/9) were diagnosed at advanced FIGO stages (stage II-IV). Case 3 could not be staged due to the absence of lymph node dissection, which precluded the determination of lymph node metastasis. Case 6 was diagnosed with MLA via biopsy and declined treatment, thus preventing staging. Seven patients underwent pelvic lymph node dissection, with postoperative pathology confirming lymph node metastasis in six cases. At the initial hospital visit, four patients presented with distant metastasis. Tables 2 and 3 summarize these findings.

**Table 2:** Demographics and clinical charaterstics in patients with MLA

	**Total (*n* ═ 11, Mean ±SD, range)**
Age (years)	11 (62.18±9.24, range, 48–74)
<60	4 (52.25±3.50, range, 48-56)
≥60	7 (67.86±5.73, range, 60–74)
BMI	
Underweight (<18.5)	0
Normal (18.5>, ≤ 25)	5
Overweight (25>, ≤30)	5
Obese (30>, ≤40)	1
Morbidly obese (≥40)	0
FIGO stage (at initial diagnosis)	
I	2
II	0
III	3
IV	4
Metastasis	
Lymph node metastasis	6
Distant metastasis	4
Survival	
Alive	11
Dead	0

Abbreviations: MLA: Mesonephric-like adenocarcinoma; BMI: Body mass index; SD: Standard deviation; FIGO: International Federation of Gynecology and Obstetrics; LNM: Lymph node metastasis; DM: Distant metastasis.

**Table 3:** Clinical charaterstics in patients, treatment and prognosis with endometrial MLA

**Case**	**Age**	**Symptom**	**Initial stage**	**Metastasis**	**Surgery**	**Adjuvant treatment**	**PFS (mo)**	**Recurrence**	**Alive/Dead**	**Follow-up time (mo)**
1	72	Postmenopausal bleeding	IIIC1	PLN	TH, BSO, PLND	No	NA	NA	Alive	36
2	64	Postmenopausal bleeding	IIIC1	PLN	TH, BSO, PLND, PALND	Yes	NA	NA	Alive	36
3	70	Lower abdominal pain	NA	NA	TH, BSO	Yes	10	Liver, lung, bone	Alive	30
4	54	Postmenopausal bleeding	IVA	PLN, PALN, bladder	TH, BSO, PLND, PALND	Yes	16	Lung, liver, spleen, brain	Alive	41
5	51	Lower abdominal pain	IVA	PLN, rectum	TH, BSO, PLND, PALND, RSE	Yes	10	Lung	Alive	15
6	74	Postmenopausal bleeding	NA	NA	NA	No	NA	NA	Alive	5
7	62	Intrauterine space-occupying lesion	IA	No	TH, BSO, PLND, PALND	Yes	27	Lung	Alive	32
8	73	Postmenopausal bleeding	IA	NA	TH, BSO	Yes	2	Multiple bone metastases	Alive	7
9	56	Cough, productive cough, blood-tinged sputum	IVB	lung	BSLLL, MLND	NA	48	Lung	Alive	56
10	60	Lower abdominal pain, Postmenopausal bleeding	IIIC1	PLN	TH, BSO, PLND	Yes	8	No	Alive	8
11	48	Pelvic mass	IVB	RO, PLN, PALN, LFT, PE	TH, BSO, PLND, PALND, OM	Yes	1	No	Alive	1

Symptom: Symptom onset to first medical visit. Adjuvant treatment: chemotherapy with or without radiation. Chemotherapy is platinum-based therapies combined with paclitaxel. Abbreviations: TH: Total hysterectomy; BSO: Bilateral salpingo-oophorectomy; PLND: Pelvic lymph node dissection; PALND: Para-aortic lymph node dissection; RSE: Rectosigmoidectomy; RO: Right ovary; LFT: Left fallopian tube; PE: Peritoneum; OM: Omentectomy; BSLLL: Basal segment of the left lower lobectomy; MLND: Mediastinal lymph node dissection.

**Figure 1.**
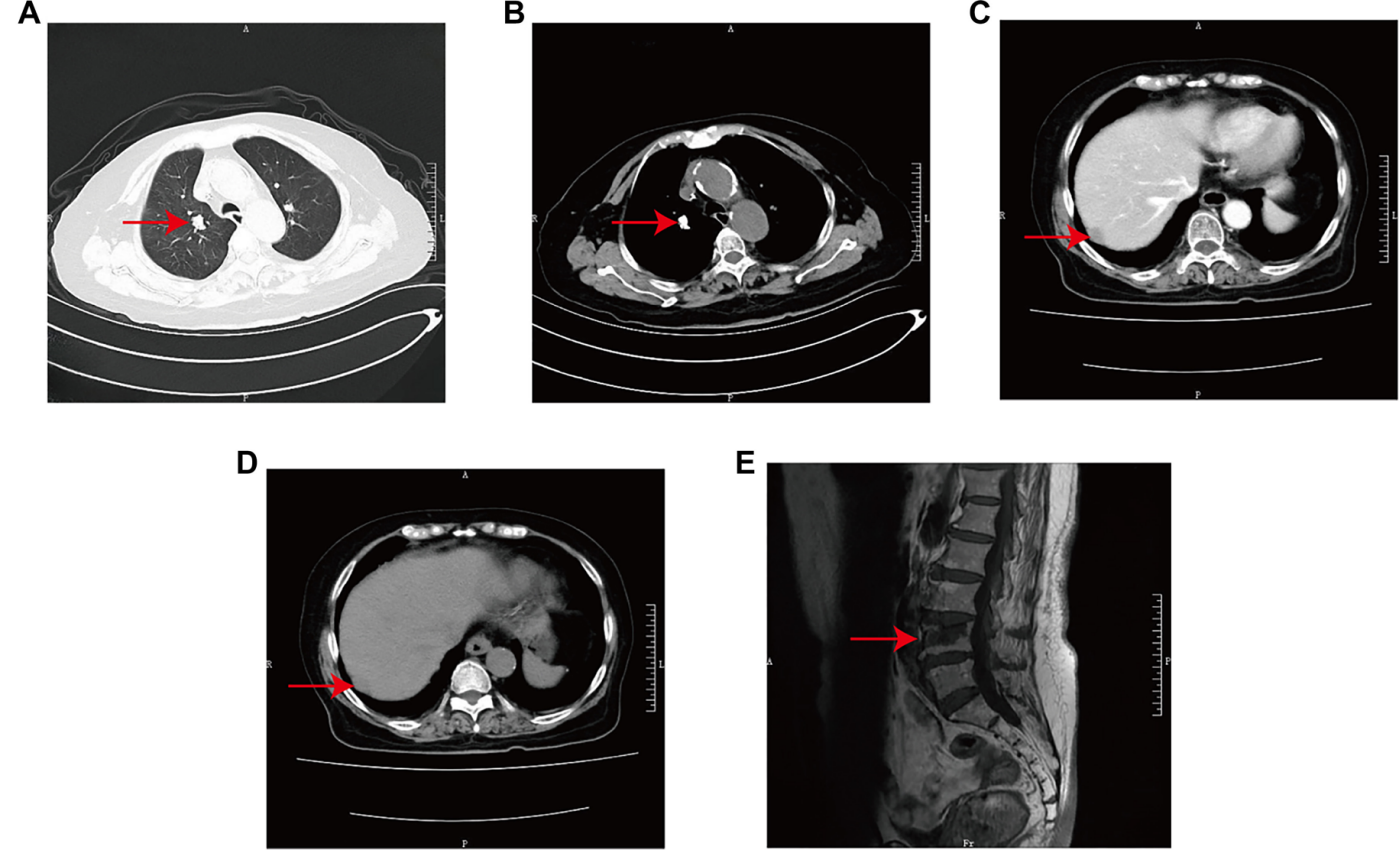
**Radiographic data from case 3—CT and MRI images.** These radiographic findings indicate the presence of metastases in the liver, lungs, and bones. (A) CT image using the lung window, showing lung metastasis (arrow). (B) CT image using the mediastinal window, also revealing lung metastasis (arrow). (C) Cross-sectional contrast-enhanced arterial phase CT of the abdomen, demonstrating liver metastasis (arrow). (D) Cross-sectional contrast-enhanced venous phase CT of the abdomen, further confirming liver metastasis (arrow). (E) Sagittal MRI images, highlighting vertebral metastasis (arrow). Abbreviations: CT: Computed tomography; MRI: magnetic resonance imaging.

**Figure 2.**
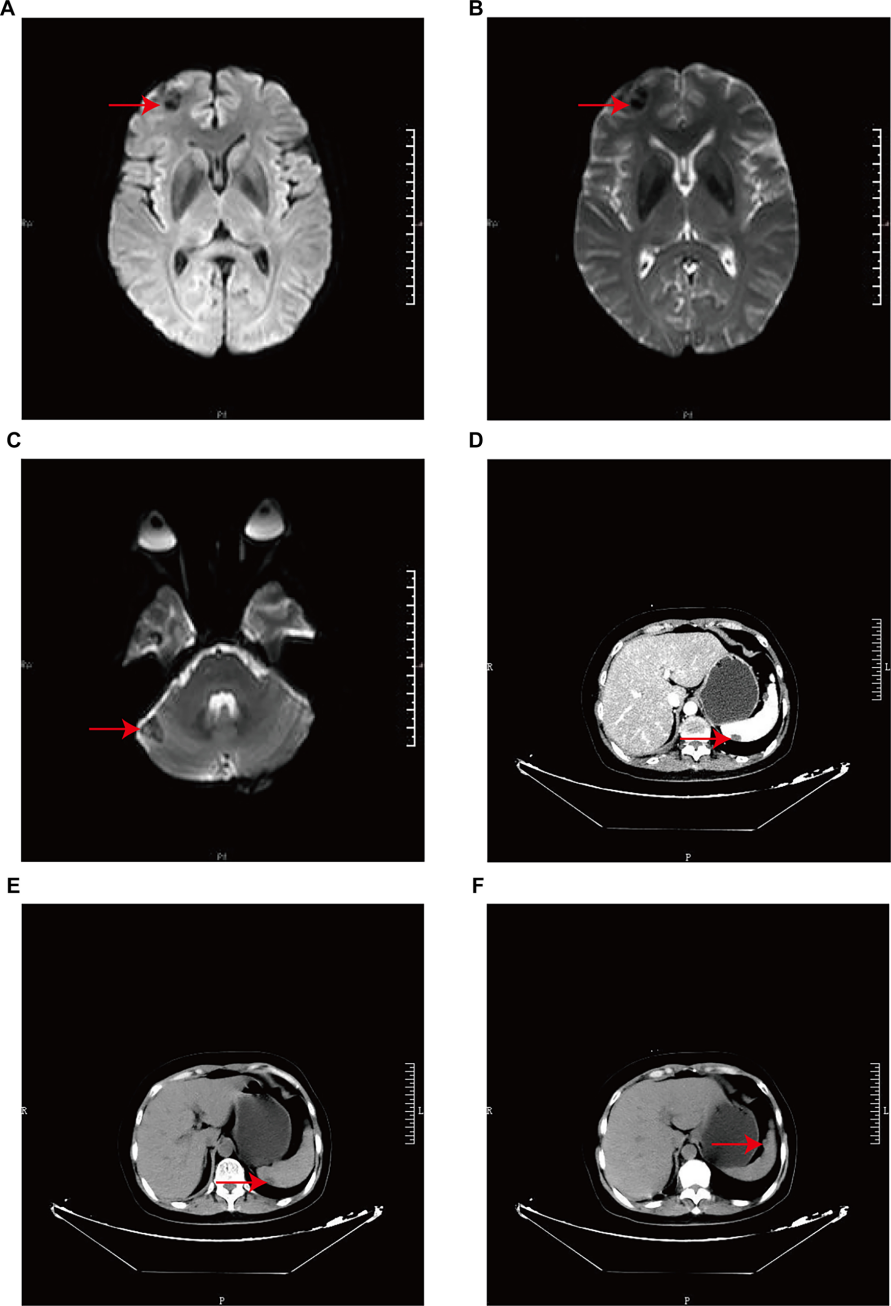
**Radiographic data from case 4. CT and MRI images.** The radiographic data illustrate the presence of metastases in both the brain and spleen. (A–C) MRI images reveal metastatic activity in the brain. (D) Cross-sectional contrast-enhanced arterial phase CT of the abdomen shows metastasis in the spleen (arrow). (E–F) Cross-sectional contrast-enhanced venous phase CT of the abdomen further confirms the presence of metastasis in the spleen (arrow). Abbreviations: CT: Computed tomography; MRI: magnetic resonance imaging.

**Figure 3.**
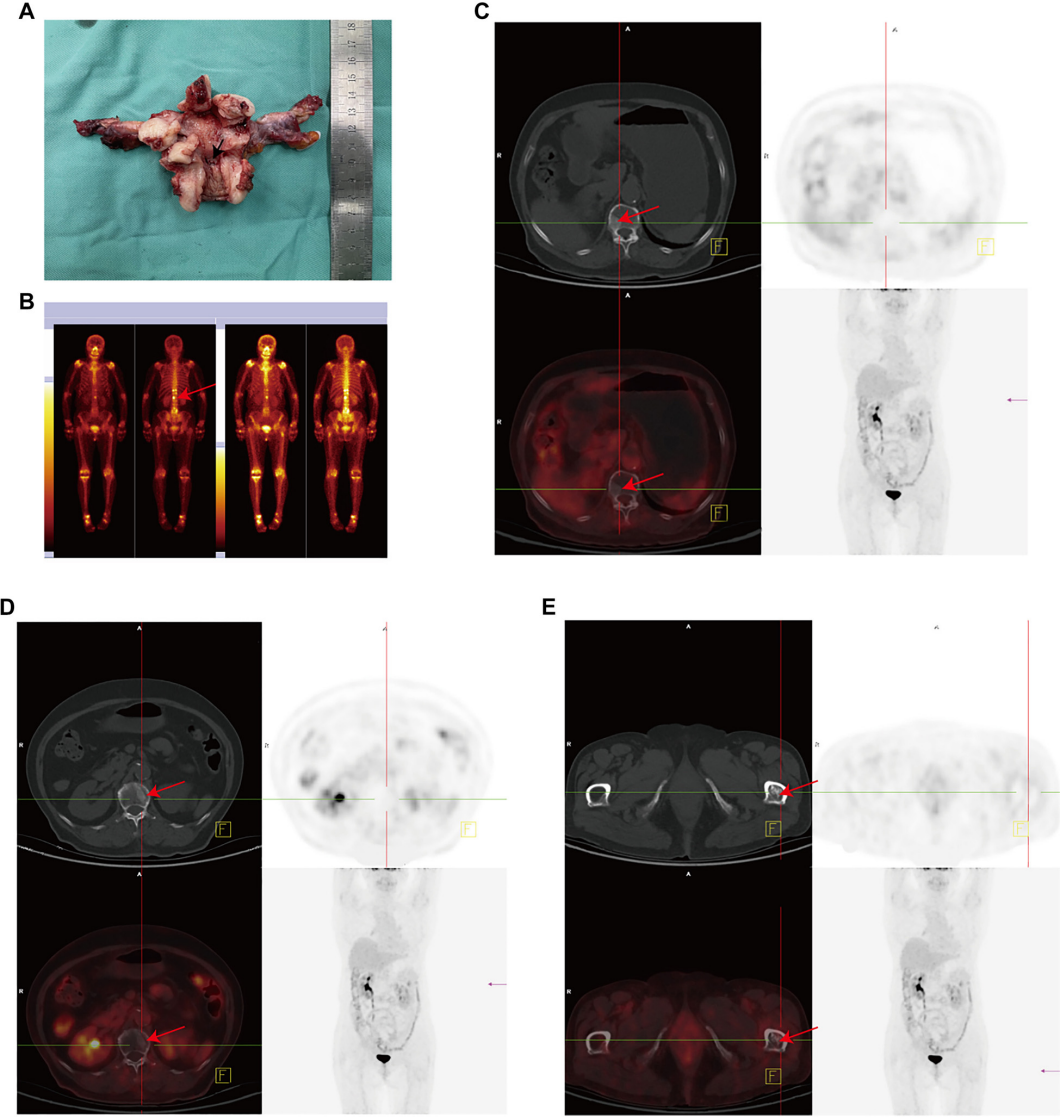
**Surgical resection specimen and radiographic data from case 8.** This figure presents SPECT and PET-CT images, illustrating that metastasis has occurred in bone. (A) The surgical procedure included hysterectomy and bilateral salpingo-oophorectomy, with lesions indicated by arrows. (B) The SPECT image reveals multiple bone metastases. (C–E) The PET-CT images further confirm the presence of multiple bone metastases. Abbreviations: SPECT: Single photon emission computed tomography; PET-CT: Positron emission tomography-computed tomography.

**Figure 4.**
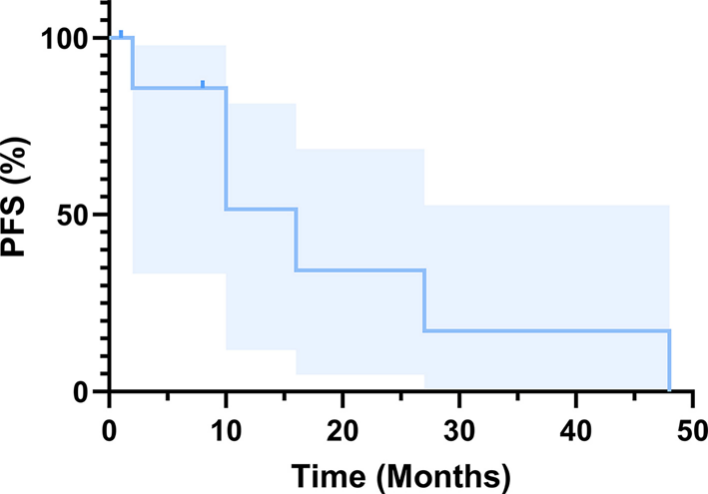
**Kaplan–Meier curve for PFS over time.** Survival analysis was performed using the Kaplan–Meier method to estimate PFS. Abbreviation: PFS: Progression-free survival.

The most common clinical manifestations included postmenopausal bleeding, lower abdominal pain, pelvic mass, cough, productive cough, and blood-tinged sputum. Of these, postmenopausal bleeding was the most prevalent, occurring in 55% (6/11) of cases. Case 9 exhibited cough, productive cough, and blood-tinged sputum due to lung metastasis. Among the patients who received lymph node dissection, 86% (6/7) showed lymph node metastasis. One patient (case 6) declined treatment, and another patient (case 9) received initial treatment at a different hospital, resulting in unclear information regarding lymph node metastasis. A total of six cases (cases 3, 4, 5, 7, 8, and 9) experienced distant recurrence during follow-up, with two cases (case 4 and case 9) presenting distant recurrence at their first visit to our hospital. Notably, case 9, which presented with lung recurrence, underwent basal segment resection of the left lower lobe and mediastinal lymph node dissection five years after the initial surgery. Multivisceral metastases were identified in three cases (cases 3, 4, and 8), with lung metastases being the most common distant metastasis (45%, 5/11). The median PFS was 16 months (95% confidence intervals: 6–26). Currently, all 11 patients are alive and well. These results are displayed in Table 3 and Figures 1–3. Kaplan–Meier curves are presented in Figure 4.

**Figure 5.**
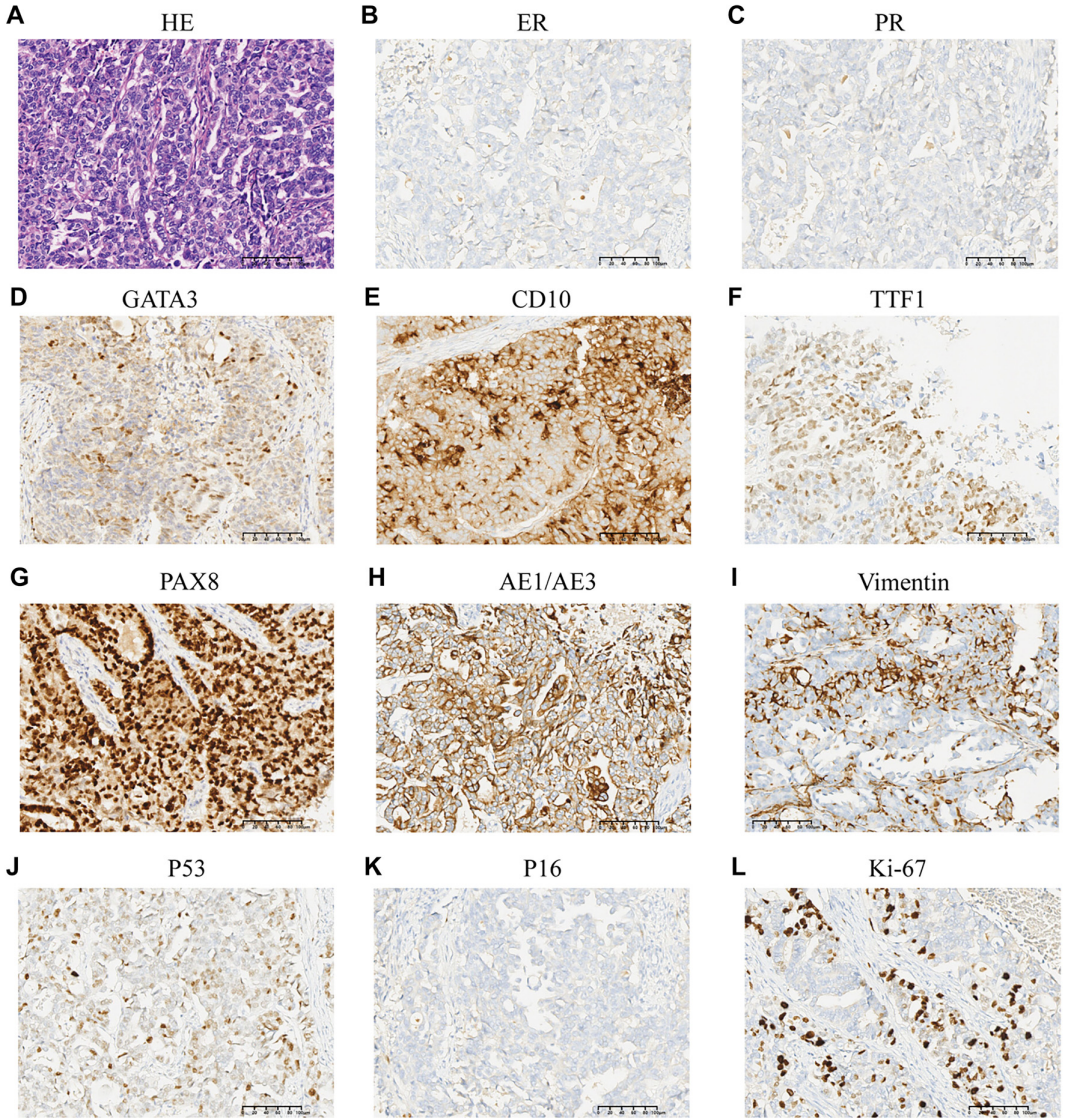
**HE staining and immunohistochemical staining of case 5.** ER and PR were negative. (A) HE staining; (B) ER negative; (C) PR negative; (D) GATA3 positive; (E) CD10 positive; (F) TTF1 positive; (G) PAX8 positive; (H) AE1/AE3 positive; (I) Vimentin positive; (J) P53 wild type; (K) P16 negative; (L) Ki-67 20% positive. Abbreviations: ER: Estrogen receptor; PR: Progesterone receptor; GATA3: GATA-binding protein 3; CD: Cluster of differentiation; TTF-1: Thyroid transcription factor-1; PAX8: Paired box 8; HE: Hematoxylin and eosin.

Plasma samples from all patients were collected for tumor biomarker analysis. Elevated levels of cancer antigen 125 (CA125) and cancer antigen 199 (CA199) were found in 55% and 36% (4/11) of cases, respectively. Additionally, vascular endothelial growth factor (VEGF) levels were elevated in 67% (4/6) of cases. Furthermore, 30% (3/10) and 27% (3/11) of cases showed elevated levels of human epididymis protein 4 (HE4) and carcinoembryonic antigen (CEA), respectively. These results are summarized in Table 4.

**Table 4:** Serum tumor biomarkers in patients at initial diagnosis

**Case**	**CA-125 U/mL** **(0.01–35)**	**CA-199 U/mL** **(0.01–37)**	**CEA ng/mL** **(0–5)**	**HE4 pmol/L** **(0–140)**	**SCC ng/mL** **(<2.5)**	**VEGF pg/mL** **(0–160)**	**AFP ng/mL** **(0–10)**
1	30.57	23.87	0.94	127	0.796	754.89 ↑	1.61
2	106.30↑	43.14↑	10.31↑	107.80	1.222	20.80	2.38
3	738.00↑	48.60↑	2.64	456↑	9.064↑	683.11↑	4.52
4	182.70↑	4.387	0.31	127.10	0.87	NA	3.70
5	151.00↑	937.00↑	5.09↑	777.00↑	0.971	330.20↑	1.30
6	29.80	8.47	0.81	114.00	1.02	NA	1.77
7	7.23	6.22	0.81	101.20	1.40	54.30	1.84
8	15.40	15.20	3.57	75.90	0.371	NA	4.61
9	10.40	15.00	1.82	NA	NA	NA	2.04
10	73.10↑	570.00↑	7.64↑	147.00↑	0.198	>800↑	3.44
11	378.00↑	3.75	0.43	87.90	NA	NA	0.97

Abbreviations: CEA: Carcinoembryonic antigen; HE4: Human epididymis protein 4; SCC: Squamous cell carcinoma antigen; VEGF: Vascular endothelial growth factor; AFP: Alpha-fetoprotein.

**Figure 6.**
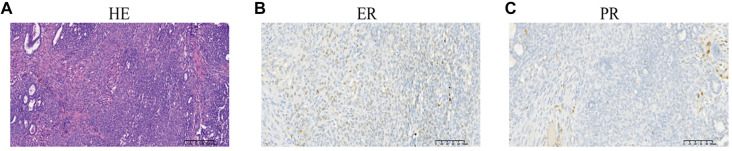
**HE staining and immunohistochemical staining of case 6.** PR was negative, and ER was weakly positive. (A) HE staining; (B) ER weakly positive; (C) PR negative. Abbreviations: ER: Estrogen receptor; PR: Progesterone receptor.

All tumors were negative for PR. The majority of cases (10/11) were also negative for ER. Most cases were positive for “mesonephric-like” markers GATA-binding protein 3 (GATA-3) (100%, 11/11), TTF-1 (91%, 10/11), and CD 10 (82%, 9/11). All patients tested positive for the “Müllerian marker” PAX-8; 90% (9/10) of patients exhibited positivity for P16; and 91% (10/11) of patients showed a wild-type P53 immunostaining pattern. P53 mutations were detected in only one case (case 3). Figure 5 illustrates the pathological features from case 5, while Figure 6 shows weak positivity for ER and negativity for PR in case 6. Among the 11 cases, five underwent KRAS mutation testing, with mutations detected in all of them (p.G12D, 2/5; p.G12A, 1/5; p.G12V, 1/5; p.G13D, 1/5). These results are presented in Table 5.

**Table 5:** Histopathological features, immunohistochemical staining, and targeted sequencing results

	**Case**										
	**1**	**2**	**3**	**4**	**5**	**6**	**7**	**8**	**9**	**10**	**11**
ER	–	–	–	–	–	W	–	–	–	–	–
PR	–	–	–	–	–	–	–	–	–	–	–
GATA3	F	+	+	P	F	F	W	P	+	Fe	F
TTF1	F	+	F	F	F	P	–	P	+	P	F
CD10	–	+	+	W	+	F	P	P	+	+	–
PAX8	+	+	+	+	+	+	+	+	+	+	+
P16	P	P	+	P	–	P	+	P	NA	P	P
P53	WT	WT	+	WT	WT	WT	WT	WT	WT	WT	WT
AE1/AE3	+	+	NA	+	+	+	+	+	+	+	+
WT1	–	–	–	–	–	NA	–	–	NA	–	–
Vimentin	P	P	+	NA	P	NA	–	P	+	–	–
Ki-67	15%+	10%+	60%+	70%+	20%+	80%+	60%+	60%+	25%+	30%+	30%+
KRAS mutation	NA	NA	p.G12D	NA	p.G12D	p.G12A	NA	NA	p.G12V	NA	p.G13D

Abbreviations: ER: Estrogen receptor; PR: Progesterone receptor; GATA3: GATA-binding protein 3; TTF-1: Thyroid transcription factor-1; CD10: Cluster of differentiation 10; PAX8: Paired box 8; WT1: Wilms tumor 1; WT: Wild type; NA: Not available; P: Partial positive; F: Focal positive; W: Weakly positive; Fe: Few positive.

## Discussion

Endometrial MLA is a rare gynecological malignancy first described in 2016 by McFarland et al. [7]. The World Health Organization (WHO) has recognized MLA as a novel pathological classification of female genital tumors [8]. Due to its rarity, the clinical behavior, pathological features, treatment options, and prognosis of endometrial MLA remain poorly understood. This single-center retrospective study aimed to examine the clinicopathological features and prognosis of 11 patients with endometrial MLA to further elucidate the molecular mechanisms underlying this condition.

A previous study reported seven cases of endometrial MLA among 237 consecutive EC cases [21], representing an incidence of 3% (7/237); another retrospective study identified an incidence of 0.7% (4/570) [9]. However, the precise incidence of endometrial MLA remains unknown. In this study, we collected data from 11 endometrial MLA cases with ages ranging from 48 years to 74 years. Although endometrial MLA can occur at any age, the majority of patients are still post-menopausal females [15, 22]. Most patients initially visited the hospital presenting with postmenopausal irregular vaginal bleeding [23]. Our data showed that 55% (6/11) of cases comprised visits for postmenopausal bleeding. Previous studies also indicated that 67% (6/9) of patients had postmenopausal bleeding [23, 24]. Additionally, over half of the cases were classified as overweight (*n* ═ 5) or obese (*n* ═ 1). Our findings suggest that obesity may be a significant risk factor for endometrial MLA; however, this hypothesis requires validation through further studies.

We diagnosed 78% (7/9) of patients at an advanced FIGO stage (stages II-IV). Staging could not be established for case 3 (which lacked lymph node dissection) and case 6 (diagnosed with MLA via biopsy and who declined treatment). In case 8, preoperative imaging analysis indicated no lymph node or distant metastasis; thus, total hysterectomy and bilateral salpingo-oophorectomy were performed without pelvic or para-aortic lymph node dissection. However, multiple bone metastases were identified two months post-surgery through positron emission tomography-computed tomography (PET-CT) (Figure 3). These findings suggest that endometrial MLA is highly malignant and predisposed to early metastasis. The median PFS was 16 months (95% confidence interval: 6–26). All patients are currently alive and in good health.

Pors et al. reported 58% (25/43) of patients with an advanced FIGO stage [10], with a median PFS of 21 months. Kim et al. [16] reported 57% (4/7) of patients at an advanced FIGO stage with a median PFS of 8 months. Elsewhere, the prognosis of 23 endometrial MLA patients was analyzed and median PFS of 18.2 months, and 70.6 months were reported in endometrial endometrioid carcinoma [25]. All these results indicated that endometrial MLA might be more aggressive than endometrioid carcinoma. To date, there is insufficient well-documented evidence to establish a definitive prognosis. Although age might be a high-risk factor for endometrial MLA, it was not significantly different for OS and PFS between stage I-II and stage III-IV [10]. Collectively, these findings indicate that endometrial MLA often presents at an advanced stage, has a poorer prognosis, and is prone to recurrence. Given its rarity and the limited number of reported cases, the outcomes of endometrial MLA remain unclear, necessitating further investigation and more robust evidence-based clinical research.

Elevated CA125 and CA199 levels were observed in 55% (6/11) and 36% (4/11) of cases, respectively. Thus, CA125 and CA199 may play a role in the early diagnosis of endometrial MLA. Prior studies have also indicated that CA125, CA199, and ubiquitin-specific protease 31 (USP31) may be associated with prognosis [20, 24, 26]. Notably, elevated VEGF was detected in 67% (4/6) of cases. However, further studies are essential to determine whether these markers can serve as reliable diagnostic and prognostic biomarkers.

Case 9 was diagnosed with endometrial cancer four years prior and underwent surgical treatment at another facility. Postoperative pathological findings confirmed uterine endometrial endometrioid carcinoma. The patient experienced lung recurrence a few months ago and received surgical intervention. Pathological findings indicated that the MLA originated in the female genital tract. It is believed that the initial pathological diagnosis may have been a misdiagnosis. Due to the absence of initial slides from the primary tumor, we are unable to reevaluate the pathological types of the primary tumor. The likelihood of a new pathological type emerging four years post-surgery is very low. Given that endometrial MLA is a rare histologic subtype recently classified as a novel pathological entity, misdiagnosis may occur due to insufficient understanding of this subtype. Based on these considerations, we believe that the initial pathological diagnosis was likely erroneous.

According to previous studies, endometrial MLA displays a variety of architectures, including ductal, tubular, papillary, retiform, solid, and sex cord-like patterns, which may contribute to misdiagnosis [10, 24, 27, 28]. Table 5 summarizes the results of immunohistochemical staining. All tested cases were positive for the “mesonephric-like” marker GATA-3 and the “Müllerian marker” PAX-8. Positive results for the “mesonephric-like” markers TTF1 and CD10 were found in 91% (10/11) and 82% (9/11) of patients, respectively. Moreover, all tested patients were PR-negative, and 91% were ER-negative. A weakly ER-positive result was noted in Case 6 (Figure 6). Consistent with the literature, ER positivity may be focal or partial in a small subset of endometrial MLA patients, indicating that ER positivity should not be used as a criterion for excluding MLA. Additionally, WT1 was negative in nearly all patients, aligning with previous findings [20]. Recent studies have indicated that papillary and ductal patterns are frequently overlooked, recommending a low threshold for performing GATA3, TTF1, and ER testing in such cases [29]. Some patients underwent testing for P53 mutations, with only one case (Case 3) exhibiting a P53 mutation. Prior research has summarized 25 cases of endometrial MLA and 19 cases of ovarian MLA, revealing a wild-type P53 expression pattern in nearly all patients, with only a few exhibiting P53 mutations [20, 30].

Furthermore, five patients underwent KRAS mutation testing, with all five (100%) presenting with KRAS mutations: p.G12D in 2/5; p.G12A in 1/5; p.G12V in 1/5; and p.G13D in 1/5. Previous research has shown that 83% (5/6) of endometrial MLA patients have KRAS mutations [16]. Additionally, KRAS mutations have been identified in 82% (23/28) of ovarian MLA patients [20]. Several larger studies have reported KRAS mutation rates ranging from 76% to 89% [5, 10, 25, 31]. These findings suggest that KRAS mutations are prevalent in endometrial MLA and may play a significant role in its onset and development.

Although most patients are diagnosed at an advanced stage, surgical resection remains the primary treatment option for those who meet surgical criteria. Postoperative chemoradiotherapy has been shown to improve prognosis. Advances in the molecular classification of EC have facilitated more precise treatment [32]. Currently, a combination of platinum and paclitaxel is the standard chemotherapeutic regimen for endometrial MLA. The common occurrence of KRAS mutations in endometrial MLA indicates that targeted KRAS therapy could enhance prognosis. However, effective KRAS-targeted therapies remain elusive due to the challenges of targeting “undruggable” mutations. Researchers have proposed that selectively inhibiting downstream targets of KRAS may represent a promising therapeutic avenue [33]. Recent studies suggest that T-cell receptor-engineered T-cell therapy targeting mutant KRAS proteins may offer another potential treatment option for patients with MLAs, with ongoing trials in other KRAS-mutated solid tumors [34]. Nevertheless, further research is warranted.

## Conclusion

This study retrospectively examined the clinicopathological features and prognosis of 11 endometrial MLA patients, revealing that endometrial MLA may be associated with a poorer prognosis. All patients who underwent KRAS mutation testing exhibited KRAS mutations, indicating that KRAS may be a promising therapeutic target. Our findings enhance the understanding of endometrial MLA; however, the study has notable limitations, including a small sample size and a retrospective, single-center design. Therefore, the applicability of these results is restricted. Future research should focus on prospective studies with larger sample sizes and multi-center designs.

## Data Availability

All data generated or analyzed in this study are included in this published article.
